# Performance of human papillomavirus E6/E7 mRNA assay for primary cervical cancer screening and triage: Population-based screening in China

**DOI:** 10.3389/fcimb.2022.935071

**Published:** 2022-08-29

**Authors:** Jing Zhang, Di Yang, Xiaoli Cui, Guangcong Liu, Zhumei Cui, Chunyan Wang, Haozhe Piao

**Affiliations:** ^1^ Department of Gynecology, Cancer Hospital of China Medical University, Liaoning Cancer Hospital and Institute, Shenyang, China; ^2^ Department of Epidemiology, Cancer Hospital of China Medical University, Liaoning Cancer Hospital and Institute, Shenyang, China; ^3^ Department of Gynecology, Affiliated Hospital of Medical College, Qingdao University, Qingdao, China; ^4^ Department of Neurosurgery, Cancer Hospital of China Medical University, Liaoning Cancer Hospital and Institute, Shenyang, China

**Keywords:** Cervical screening, HPV E6/E7 mRNA, HR-HPV prevalence, cytology, triage

## Abstract

**Objective:**

Cervical cancer screening is very important in the prevention and treatment of cervical cancer. In China, the cervical screening strategy needs to be improved. To explore a suitable cervical screening strategy in China, we evaluated the performance of the human papillomavirus (HPV) E6/E7 mRNA (Aptima HPV (AHPV)) assay in primary screening and different triage strategies for women undergoing routine cervical screening.

**Methods:**

A total of 10,002 women aged 35 to 65 years of age were recruited in Liaoning Province and Qingdao City, China. Specimens were tested by liquid-based cytology (LBC) and the AHPV assay, and women who tested positive on any test were referred for colposcopy. Genotyping was performed on all high-risk HPV (HR-HPV)-positive samples. Test characteristics were calculated based on histological review.

**Results:**

We identified 109 women with high-grade squamous intraepithelial lesion or worse (HSIL+), including six with cervical cancer. The sensitivity of AHPV was clearly higher than that of LBC (92.7 [95% CI: 87.2, 97.2] *vs*. 67.9 [95% CI: 59.6, 76.1], *p* < 0.001). The specificity of AHPV was 93.0 (95% CI: 92.5, 93.5), which was lower than that of LBC (95.2 [95% CI: 94.8, 95.6], *p* < 0.001). There was no statistical difference between the positive predictive value of AHPV and LBC (13.5 [95% CI: 11.2, 16.2] *vs*. 14.3 [95% CI: 11.4, 17.6], *p* = 0.695). The difference of area under the curve (AUC) values between the AHPV test (0.928 [95% CI: 0.904, 0.953]) and LBC test (0.815 [95% CI: 0.771, 0.860]) in detecting HSIL+ was statistically significant (*p* < 0.001). Finally, among the three triage strategies, both the sensitivity (73.4 [95% CI: 65.1, 81.7]) and AUC (0.851 [95% CI: 0.809, 0.892]) of AHPV genotyping with reflex LBC triage were the greatest.

**Conclusion:**

In summary, the AHPV assay is both specific and sensitive for detecting HSIL+ and may be suitable for use in primary cervical cancer screening in China. AHPV genotyping with reflex LBC triage may be a feasible triage strategy.

## Introduction

Cervical cancer is one of the main malignant tumors threatening women’s health. The incidence and mortality rates of cervical cancer in China have increased year by year in the past 20 years, with a trend for this disease to increasingly affect younger women (Yu and Chen, 2015; Chen et al., 2016; Sung et al., 2021) . Persistent infection with high-risk human papillomavirus (HPV) is the main pathogenic factor for cervical cancer. The prevention of cervical cancer has been widely performed globally. However, cervical cancer vaccination started late in China, and the vaccinated population coverage is low. It is thus important to improve our current screening systems. The performance of traditional Pap smear in developing countries and regions is not satisfactory, with a sensitivity of only 30%–40% (Gay et al., 1985). Liquid-based cytology has improved its performance, but the number of cytopathologists in China remains insufficient, and the diagnostic skills are uneven, which hinders the popularization of this technology for routine screening. The visual inspection with acetic acid and Lugol iodine (VIA/VILI) screening method does not depend on specific equipment and is simple and inexpensive to operate but has low sensitivity (40%–60%) (Denny et al., 2005; Wei et al., 2012). In view of the oncogenic etiology, HPV testing could serve as an accurate means of detecting women at risk of cervical cancer. High-risk HPV (HR-HPV) testing was recommended in Europe in 2008 for primary cervical cancer screening in women older than 25 years, and in April 2014, the United States Food and Drug Administration (FDA) approved the Cobas 4800 HPV-DNA test for primary cervical cancer screening in women older than 25 years (Huh et al., 2015). The latest guidelines published by the WHO in 2021 also recommend HPV for primary screening (World Health Organization, 2021). The results of HPV testing were shown to be relatively accurate and consistent irrespective of the assays used, and HPV primary screening increased the rate of detection of cervical intraepithelial neoplasia lesions of grade 2 or more (CIN2+) by 25% (Zhang et al., 2021). Primary HPV screening has the advantage of high sensitivity but lacks specificity. The four HPV tests currently approved by the FDA include three DNA-based assays and one RNA-based assay. The detection of HPV E6/E7 mRNA could theoretically have higher specificity (Iftner et al., 2015). The E6/E7 oncogenes are well known to play critical roles in the development of cervical cancer. Since E6/E7 overexpression occurs after the integration of HPV into the genome, direct testing of HR-HPV E6/E7 in cervical samples may turn out to be more specific than HR-HPV-DNA testing in detecting high-grade cervical lesions (Ge et al., 2018). Upon comparison with HPV-DNA testing using the non-inferiority score test, the HPV E6/E7 mRNA assay met the cross-sectional clinical and reproducibility criteria of the international guidelines for HPV test requirements for cervical screening in the detection of CIN2+ (Heideman et al., 2013). At present, several studies, including those from Shenzhen, China (Wu et al., 2010), Henan Province, China (Zhang et al., 2020b), Wenzhou, China (Pan et al., 2019), and Tehran, Iran (Mousavi et al., 2020), also confirmed the efficacy and feasibility of the HPV E6/E7 mRNA assay. However, these studies were limited by their small sample size and were mostly hospital-based studies. There is thus a need for a large prospective population-based screening study to assess the performance of the HPV E6/E7 mRNA assay in cervical cancer screening in China. Moreover, seldom did previous reports evaluate possible triage strategies in Aptima HPV (AHPV)-positive women (Wang et al., 2019).

Therefore, in this study, we analyzed and assessed the performance of different primary screening schemes and various triage strategies by conducting a large cross-sectional study of population-based cervical cancer screening in Liaoning Province and Qingdao City in China, in order to advance the level of prevention and treatment of cervical cancer in China.

## Materials and methods

### Study population

The study population was recruited from Liaoning Province (Shenyang City for an urban population and Benxi County and Sujiatun District for rural populations) and Qingdao City (for an urban population) between April 2018 and December 2021. The criteria for inclusion in the community screening population were as follows: resident population with household registration (living locally for more than 3 years) in the screening area, aged 35–65 years, no severe organ dysfunction or mental illness, volunteering to participate, and being able to complete the questionnaire. Meanwhile, the exclusion criteria were as follows: women with a history of hysterectomy, pelvic radiation therapy, pregnancy, or lactation, and those with other serious medical and surgical conditions under treatment. This study was approved by the ethics committee of Liaoning Cancer Hospital (approval number: 20180106).

### Study design

In terms of the study design, upon enrollment, a single cervical specimen was collected from all participants using a Cytobrush and suspended in PreservCyt collection medium (Hologic Inc., Marlborough, MA, USA), in accordance with the manufacturer’s instructions. Each specimen was used for liquid-based cytology (LBC) and the AHPV assay (Hologic, San Diego, CA, USA). Participants who had atypical squamous cells of undetermined significance (ASC-US) or a worse cytologic diagnosis and/or were HPV-positive on either assay were referred for colposcopy and biopsy. Colposcopy was performed by specialized colposcopists at a cervical lesion clinic. Colposcopy-guided biopsy was performed if abnormal epithelium was observed. If the colposcopy assessment was inadequate, random biopsies at 3, 6, 9, and 12 o’clock positions in the cervix and endocervical curettage (ECC) were performed. Patients who had an ASC-US or low-grade squamous intraepithelial lesion (LSIL) cytology test result and showed no visible lesion during colposcopy at the first visit were not subjected to biopsy and were considered to have a histological status of “no HSIL”. If cervical cancer was suspected during sampling, a cervical biopsy was performed immediately. Women with negative co-screening results were considered to have a histological status of “no HSIL”. The biopsy results were categorized into the following three general groups: benign (including no pathological alteration and benign or reactive lesions), low-grade squamous intraepithelial lesions (LSIL, CIN 1, and HPV effect), and high-grade cervical lesions or worse (HSIL+). All CIN 2 lesions were confirmed by immunohistochemical staining for p16 and Ki-67.

### Liquid-based cytology

All samples were first analyzed by ThinPrep^®^ LBC (Hologic Inc., USA). LBC results were evaluated according to the 2014 Bethesda System.

### Human papillomavirus testing and genotyping

The LBC specimens were tested under blinded conditions with the Aptima^®^ HPV assay (Gen-Probe; Hologic, San Diego, CA), an FDA-certified HPV E6/E7 mRNA assay that detects 14 HR-HPV types (HPV16, 18, 31, 33, 35, 39, 45, 51, 52, 56, 58, 59, 66, and 68). All HR-HPV-positive samples were further genotyped by the Aptima^®^ HPV 16 18/45 Genotype (GT) assay (AHPV-GT) (Gen-Probe; Hologic, San Diego, CA, USA). The AHPV-GT can detect HPV16 and a subset of HPV18 and HPV45 cases (Wang et al., 2019). Detection and result reporting were performed by professional technicians in accordance with the manufacturer’s instructions.

### Data analysis

**Figure 1:**
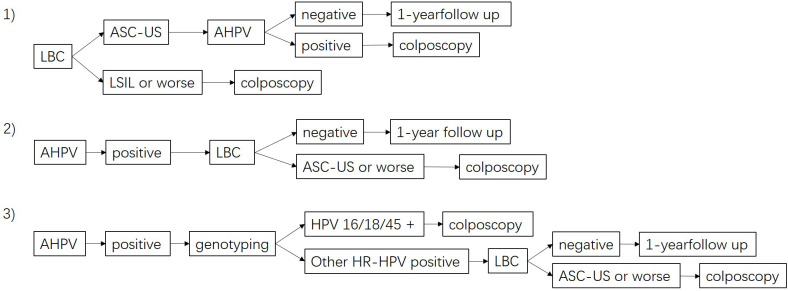
Flow diagram of the different triage strategies. 1) LBC-AHPV triage, 2) AHPV-LBC triage, and 3) AHPV genotyping with reflex LBC triage. LBC, liquid-based cytology; AHPV, Aptima human papillomavirus.

Positivity in primary screening with LBC was defined with ASC-US+. Positivity in primary screening with AHPV was defined as positivity for any HR-HPV infection. Positivity in combined screening with co-testing (LBC+AHPV) was defined as positivity for either ASC-US+ or HR-HPV infection. Three kinds of possible triage strategies are shown in **Figure 1**. 1) LBC-AHPV: patients with an LBC test result with ASC-US were referred for colposcopy if the AHPV test was positive, and patients with an LBC test result with LSIL or worse were referred directly. 2) AHPV-LBC: AHPV test-positive cases were referred if the LBC test gave a result of ASC-US or worse. 3) AHPV genotyping with reflex LBC triage: AHPV-positive cases were further tested by HPV genotyping and referred if HPV16/18/45-positive, or if positive for other HR-HPV genotypes with an LBC test result of ASC-US or worse. Histological confirmation of HSIL+ served as the clinical observation endpoint. The sensitivity, specificity, positive predictive value (PPV), negative predictive value (NPV), Youden’s index, and the area under the receiver operating characteristic (ROC) curve (AUC) were determined in line with standard definitions when comparing different diagnostic tests. The 95% confidence intervals (CIs) of proportions were calculated. Sensitivity and specificity were compared using McNemar’s test for paired data, and Pearson’s chi-square test for comparing predictive values of diagnostic tests was used to compare PPVs and NPVs. AUC was compared by the Delong test. The number of referred colposcopies to detect one case of HSIL+ was calculated as a measure of the screening efficiency of the screening method. Age was presented with median and interquartile range, as age was not normally distributed. Differences between categorical variables were compared by chi-squared (χ^2^) tests. Statistical analysis was performed using SPSS 22.0, and *p* < 0.05 was considered statistically significant.

## Results

### Characteristics of the study population

**Figure 2:**
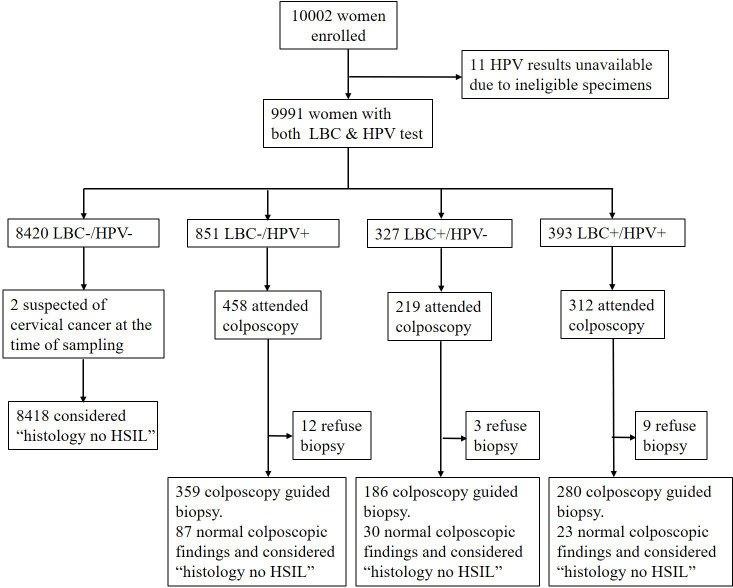
Flow diagram of the study population.

A total of 10,002 eligible women were recruited in this study within 34 communities between April 2018 and December 2021. They provided both a specimen and a completed questionnaire. The median age of the participants was 49 years (interquartile range, 44–55). Of these, 7,978 (79.8%) were from Liaoning Provence, and 2,024 (20.2%) were from Qingdao City; 5,994 (59.9%) were from an urban population and 4,008 (40.1%) from a rural one. Overall, 7,789 (77.9%) had never previously participated in cervical cancer screening. However, HPV results were not available in 11 women due to ineligible specimens. The baseline characteristics of the 9,991 women with both cytology and HPV screening results are shown in **Table 1**. Among them, 720 (7.2%) women had positive cytology results (ASC-US or worse), and 1,244 (12.5%) women were infected with HPV. Of the 1,571 women who tested positive on any screening test, 989 (63.0%) underwent colposcopy, and 965 (97.6%) had either adequate negative colposcopy findings or an adequate biopsy specimen. Two women were suspected of having cervical cancer at the time of sampling, so a cervical biopsy was also performed at that time (**Figure 2**). HPV genotyping results were available in 1,213 women. Among them, 184 (15.2%) were HPV16-positive, 65 (5.4%) were HPV18/45-positive, and 980 (80.8%) were positive for other HR-HPV types, including 16 women with multiple infections of these three groups of HPV genotypes.

**Table 1:** The baseline characteristics of the 9,991 women with both LBC and AHPV test results.

		N (%)
Age	35–44	2,603 (26.1)
	45–55	5,225 (52.3)
	56–65	2,163 (21.6)
Age of first sex	≤19 years	236 (24.8)
	>19 years	9,262 (75.2)
No. of sexual partners	≥2	613 (6.4)
	1	8,893 (93.6)
No. of parturitions	>1	1,043 (12.1)
	≤1	7,557 (87.9)
Smoking	Yes	619 (6.2)
	No	9,371 (93.8)
Cytology	Positive	720 (7.2)
	Negative	9,271 (92.8)
AHPV	Positive	1,244 (12.5)
	Negative	8,747 (87.5)

LBC, liquid-based cytology; AHPV, Aptima human papillomavirus.

### Detection rate of high-grade squamous intraepithelial lesion or worse

A total of 109 women with HSIL+ (1.1% of the cohort) were identified, among whom six (6.0/100,000 of the cohort) had confirmed cervical cancer. The rates of HSIL+ detection did not differ significantly between the urban and rural cohorts (1.0% *vs*. 1.2%, χ^2^ = 0.419, *p* = 0.556).

In addition, the rate of HSIL+ detection was not correlated with the menopausal status of women (χ^2^ = 0.000, *p* = 0.982). The rate of HSIL+ detection in HPV-negative women was significantly lower than that of cytologic normality (0.1% *vs*. 0.4%, χ^2^ = 16.294, *p* < 0.001). The rate of HSIL+ detection was significantly higher in HPV16-positive women than in HPV18/45-positive women and those positive for other high-risk genotypes (χ^2^ = 44.685, *p* < 0.001). HSIL+ was detected significantly more often in women infected with HPV16/18/45 than in women with a cytological status of ASC-US+ (35.5% *vs*. 14.2%, χ^2^ = 11.769, *p* = 0.001) (**Table 2**).

**Table 2:** Distribution of histological diagnosis results stratified by menopause status, cytology, HPV, and genotyping test results (N [%]).

	Normal	LSIL	HSIL+	Total	χ^2^	*p*
**Total**	8,987 (95.8)	289 (3.1)	109 (1.2)	9,385		
**Menopause status**						
Premenopause	4,861 (95.9)	150 (3.0)	59 (1.2)	5,070	0.000	0.982
Menopause	4,126 (95.6)	139 (3.2)	50 (1.2)	4,315		
**Cytology**						
NILM	8,666 (97.7)	165 (1.9)	35 (0.4)	8,866	16.294*	<0.001*
ASCUS	257 (74.9)	65 (19.0)	21 (6.1)	343		
ASC-H	14 (35.9)	10 (25.6)	15 (38.5)	39		
LSIL	37 (36.6)	47 (46.5)	17 (16.8)	101		
HSIL	0 (0.0)	2 (10.0)	18 (90.0)	20		
AGC	13 (81.3)	0 (0.0)	3 (18.8)	16		
**HPV test**						
Negative	8,575 (99.3)	53 (0.6)	8 (0.1)	8,636		
Positive	412 (55.0)	236 (31.5)	101 (13.5)	749		
HPV16	37 (35.2)	32 (30.5)	36 (34.3)	105	44.685	<0.001
HPV18/45	30 (68.2)	11 (25.0)	3 (6.8)	44		
Other HR-HPV	341 (57.7)	188 (31.8)	62 (10.5)	591		

HPV, human papillomavirus; LSIL, low-grade squamous intraepithelial lesion; HSIL, high-grade squamous intraepithelial lesion; HR-HPV, high-risk HPV.

*Comparison of the detection rate of HSIL+ between cytology-negative women and HPV-negative women. NILM, negative for intraepithelial lesion or malignancy; ASC-US, atypical squamous cell of undetermined significance; ASC-H, atypical squamous cells, HSIL cannot be excluded; AGC, atypical glandular cells.

### Comparison of performance of different primary screening tests, combined screening, and triage strategies

**Figure 3:**
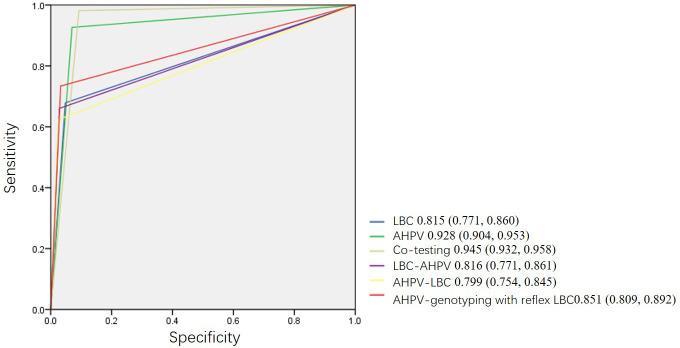
Receiver operating characteristic curves (ROC) of different primary screening tests, combined screening, and triage strategies.

A comparison of the performance of the primary screening tests and combined screening for the detection of HSIL+ is shown in **Table 3**. The sensitivity of AHPV was 92.7 (95% CI: 87.2, 97.2), which was significantly higher than that of LBC at 67.9 (95% CI: 59.6, 76.1) (*p* < 0.001). This difference was associated with LBC missing 27 (24.8%, 27/109) cases of HSIL+ in the cohort. However, the specificity of AHPV was lower than that of LBC (93.0 [95% CI: 92.5, 93.5] *vs*. 95.2 [95% CI: 94.8, 95.6], *p* < 0.001). The difference between PPVs of AHPV and LBC had no statistical significance (13.5 [95% CI: 11.2, 16.2] *vs*. 14.3 [95% CI: 11.4, 17.6], *p* = 0.695). Compared to the LBC or AHPV primary screening, although co-testing had the highest sensitivity of 98.2 (95% CI: 95.4, 100.0) (*p* < 0.001, *p* = 0.03 respectively), it had the lowest specificity of 90.8 (95% CI: 90.2, 91.3) (both *p* < 0.001). The AUC of co-testing was the greatest at 0.945 (95% CI: 0.932, 0.958), followed by AHPV at 0.928 (95% CI: 0.904, 0.953). The difference in the AUC of co-testing and the AHPV test for HSIL+ was not statistically significant (*p* = 0.1407); however, the AUC of the AHPV test was significantly greater than that of the LBC test (0.815 [95% CI: 0.771, 0.860], *p* < 0.001) (**Figure 3**).

**Table 3:** Comparison of performance of different primary screening tests and different triage strategies.

Primary screening tests	No. of HSIL+	The rate of referred to colposcopy (%)	Sensitivity (95% CI)	Specificity (95% CI)	PPV (95% CI)	NPV (95% CI)		Youden’s index (95% CI)	AUC (95% CI)	*p*
LBC	74	7.2	67.9 (59.6, 76.1)	95.2 (94.8, 95.6)	14.3 (11.4, 17.6)	99.6 (99.4, 99.7)		0.63 (0.54, 0.72)	0.815 (0.771, 0.860)	<0.001^*^
AHPV	101	12.5	92.7 (87.2, 97.2)	93.0 (92.5, 93.5)	13.5 (11.2, 16.2)	99.9 (99.8, 100.0)		0.86 (0.80, 0.91)	0.928 (0.904, 0.953)	
Co-testing	107	15.7	98.2 (95.4, 100.0)	90.8 (90.2, 91.3)	11.1 (9.7, 10.9)	100.0 (99.9, 100.0)		0.89 (0.86, 0.91)	0.945 (0.932, 0.958)	0.1407^#^
LBC-AHPV	72	4.4	66.1 (56.9, 75.2)	97.2 (96.8, 97.5)	21.6 (17.7, 26.4)	99.6 (99.4, 99.7)		0.63 (0.54, 0.73)	0.816 (0.771, 0.861)	0.0564^**^
AHPV-LBC	68	3.9	62.4 (53.2, 71.6)	97.5 (97.1, 97.8)	22.4 (18.0, 27.6)	99.5 (99.4, 99.7)		0.60 (0.50, 0.69)	0.799 (0.754, 0.845)	0.0006^##^
AHPV genotyping with reflex LBC	80	5.5	73.4 (65.1, 81.7)	96.7 (96.4, 97.1)	20.9 (17.0, 25.4)	99.7 (99.5, 99.8)	0.70 (0.62, 0.79)		0.851 (0.809, 0.892)	

LBC-AHPV, LBC tests with ASCUS were referred if AHPV test was positive, and LBC test with LSIL or worse was referred directly. AHPV-LBC, AHPV test-positive cases were referred if LBC test with ASCUS or worse. AHPV genotyping with reflex LBC, AHPV-positive cases were further tested by HPV genotyping and referred to colposcopy if HPV16, 18/45-positive, or if other HR-HPV genotypes positive with LBC test ASCUS or worse.

HSIL, high-grade squamous intraepithelial lesion; AUC, area under the receiver operating characteristic curve; PPV, positive predictive value; NPV, negative predictive value; LBC, liquid-based cytology; AHPV, Aptima human papillomavirus; LSIL, low-grade squamous intraepithelial lesion.

^*^Comparison of AUC between LBC and AHPV.

^#^Comparison of AUC between AHPV and co-testing.

^**^Comparison of AUC between LBC-AHPV and AHPV genotyping with reflex LBC.

^##^Comparison of AUC between AHPV-LBC and AHPV genotyping with reflex LBC.

In **Table 3**, three triage strategies were analyzed. Based on the primary screening test being either the LBC test or AHPV test, triage using LBC, AHPV, or genotyping would yield higher PPVs, but a smaller proportion of cases would be referred to colposcopy. The sensitivity of AHPV genotyping with reflex LBC triage was the highest at 73.4 (95% CI: 65.1, 81.7). The difference between the sensitivity of AHPV genotyping with reflex LBC triage and AHPV-LBC triage (62.4 [95% CI: 53.2, 71.6]) was statistically significant (*p* < 0.001). The specificity of AHPV genotyping with reflex LBC triage at 96.7 (95% CI: 96.4, 97.1) was lower to that of AHPV-LBC triage at 97.5 (95% CI: 97.1, 97.8) (*p* < 0.001). The AUC of AHPV genotyping with reflex LBC triage strategy was the greatest (0.851 [95% CI: 0.809, 0.892]), which was significantly greater than that of AHPV-LBC triage (0.799 [95% CI: 0.754, 0.845], *p* = 0.0006), and there was a tendency that the AUC of AHPV genotyping with reflex LBC triage is slightly greater than that of LBC-AHPV triage (0.816 [95% CI: 0.771, 0.861], *p* = 0.0564) (**Figure 3**).

### Screening efficiency

**Figure 4:**
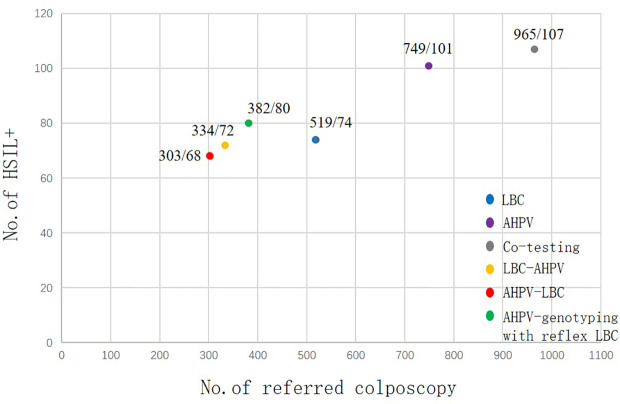
Screening efficiency of the different primary screening and three triage strategies for HSIL+. HSIL, high-grade squamous intraepithelial lesion.

With AHPV, 749 women were referred for colposcopy, among whom 101 HSIL+ cases were detected. Therefore, seven to eight women (749/101) need to be referred for each case of HSIL+ detected. Moreover, for each case of HSIL+ detected in LBC, seven women (519/74) need to be referred, so AHPV is nearly equivalent to LBC in terms of screening efficiency (**Figure 4**).

## Discussion

This study evaluated and compared the performance of HPV E6/E7 mRNA (AHPV) assay, liquid-based cytology (LBC), and co-testing as primary screening and three different triage strategies in a routine community screening cohort of 10,002 women in China. We found that HPV E6/E7 mRNA had high sensitivity and good specificity in detecting high-grade cervical precancerous lesions.

Large population-based studies in China previously reported HPV prevalence ranging from 9.9% to 27.5% (Li et al., 2013). In this study, the prevalence of HPV infection was 12.5%, which was at the low end of the reported range of HPV prevalence in China. In terms of the reasons for this low rate, first, Liaoning Province and Qingdao City are not areas with a high HPV prevalence. Second, the population in this study was population-based, while some previous studies were instead hospital-based. Finally, it was found that the rate of positivity in the HPV E6/E7 mRNA assay was lower than that in the HPV-DNA assay (Mousavi et al., 2020).

HPV testing has the advantages of being objective, the results being obtained in a short time and being easy to repeat. HPV testing can detect precancerous cervical lesions earlier than cytology. A negative result of HR-HPV testing was reported to predict a lower risk of future CIN2+ and could enable screening to be performed less regularly (Ogilvie et al., 2012; Ronco et al., 2014; Zhang et al., 2021). In theory, HPV E6/E7 mRNA is produced after the genomic integration of HPV viral genes and might represent a state of active HPV infection, so the detection of HPV E6/E7 mRNA transcripts may provide greater specificity for CIN2+ (Zhang et al., 2017). HPV E6/E7 mRNA assay has been focused on in recent years, and several studies have compared its performance to that of the HPV-DNA assay for cervical cancer screening. For example, a study enrolling 9,451 women aged 30–60 years attending routine cervical cancer screening in Germany compared an RNA-based test (AHPV) and a DNA-based test (HC2), finding no statistically significant difference in sensitivity in detecting CIN2+ (*p* = 0.180) or CIN3+ (*p* = 0.0625) lesions between them. The specificity (<CIN2) and positive predictive value (CIN2+) of the AHPV test were significantly higher than those of the HC2 test (*p* < 0.001) (Iftner et al., 2015). The RNA-based assay detected actively infected cells, whereas DNA-based assays, such as HC2, could not distinguish between intracellular and extracellular viral DNA, leading to the results potentially being affected by contamination with extracellular viral particles. Consistent with this, the slightly lower sensitivity of the RNA-based HPV test in detecting CIN2+ was reported in other earlier studies (Monsonego et al., 2011). Moreover, more recent reports demonstrated equal (Ratnam et al., 2011; Nieves et al., 2013) or higher (Wu et al., 2010; Monsonego et al., 2012; Kuroki et al., 2021) sensitivity of the AHPV test compared with that of the HC2 test. In addition, compared to the Cobas HPV test, AHPV and GT demonstrated significantly higher specificity and PPV (Ge et al., 2018). After long-term follow-up, the future risk of HSIL+ in women with a negative HPV E6/E7 mRNA test result was found to be quite low, as was that of women with negative results in DNA-based assays (Reid et al., 2015; Iftner et al., 2019). In this study, HPV E6/E7 mRNA testing showed good performance in population-based cervical cancer screening with high sensitivity of 92.7 (95% CI: 87.2, 97.2) and high specificity of 93.0 (95% CI: 92.5, 93.5). Therefore, HPV E6/E7 mRNA testing could be suitable for the primary screening of cervical cancer.

Current practice in China mainly involves annual cytological screening. The detection rate of precancerous cervical lesions and cervical cancer by cytology testing was reported to be only 124.87–491.03/100,000 in China (Song et al., 2015; Song et al., 2021). However, the age-standardized prevalence of CIN2+ lesions was 2.7% among women in rural China and 1.3% among women in urban China, which are high among Asian women. Therefore, the poor sensitivity of cytology demands the development of more accurate screening approaches. Strategies for improving the detection of CIN2+ cases have been assessed for the HPV E6/E7 mRNA assay based on the primary screening test being cytology (Monsonego et al., 2011; Ratnam et al., 2011; Monsonego et al., 2012; Nieves et al., 2013). In this study, the rate of HSIL+ detection in the co-testing screening was 1.1% in the total population, 1.0% in the urban population, and 1.2% in the rural population. The detection rate in the urban population was close to the prevalence of CIN2+ in women in urban China, but the detection rate in the rural population still needs to be improved. In this study, HSIL+ was detected at a rate of 1.0% by RNA-based HPV assay screening alone but at 0.74% by LBC screening alone. The AUC value of AHPV screening alone of 0.928 (95% CI: 0.904, 0.953) was significantly higher than that of LBC screening alone of 0.815 (95% CI: 0.771, 0.860; *p* < 0.001). Although the AUC value of AHPV screening alone was slightly lower than that of co-testing screening, the difference was not statistically significant (*p* = 0.1407). Co-testing screening might only be applicable in economically developed parts of China because of its high cost. Considering the screening efficiency, this study also implied that only slightly more women would need to be referred to detect one HSIL+ case (eight women with the AHPV assay versus seven with cytology alone), but a larger proportion (36.5%) of additional HSIL+ cases would be identified (101 cases for the AHPV assay versus 74 for cytology alone), compared with the use of AHPV with cytology as a primary screening test. Therefore, compared with the cytology test, the HPV E6/E7 mRNA assay may be a better option in primary cervical cancer screening in China.

HPV E6/E7 mRNA assay was an effective triage method in women with a cytological result of ASC-US (Stoler et al., 2013). In this study, compared with the LBC test alone, with the use of AHPV as triage for the cytology test, although the sensitivity decreased slightly (67.9%, 66.1%), the specificity increased (95.2%, 97.2%), the colposcopy referral decreased (7.2%, 4.4%), and the AUC value of the two methods was close (0.815, 0.816). Moreover, with the use of AHPV as triage for the cytology test, 334 women would have been referred, with 72 cases of HSIL+ being detected immediately. This means that the number of referrals may be drastically reduced by more than 35%, while the number of detected HSIL+ cases remained unchanged.

Although the sensitivity of the AHPV alone strategy reached 92.7%, the colposcopy referral was achieved at 12.5%, and its PPV for HSIL+ was 13.5%, consistent with the literature at 6.3%–21.1% in cervical cancer primary screening (Cuzick et al., 2013; Iftner et al., 2015). Primary HPV testing with triage methods was necessary. There were few studies on AHPV-based screening triage strategy. Wang et al. reported the sensitivity of AHPV-positive women triaged with cytology was 59.5% (Wang et al., 2019). In this study, the sensitivity of AHPV-LBC triage was 62.4%. AHPV with cytology-only triage had relatively low sensitivity. On the one hand, the limited sensitivity of cytology might be a detriment to HPV testing sensitivity, especially in places where strict quality assurance cannot be ensured (Almonte et al., 2020). On the other hand, AHPV-LBC triage would miss to detect HSIL+ in HPV-positive women with normal cytology, especially in HPV16/18-positive women with normal cytology (Wentzensen et al., 2016). HPV genotyping triage provided better risk stratification and required fewer women to attend close testing (Zhao et al., 2021). In this study, the rate of referred to colposcopy for AHPV genotyping with reflex LBC triage was only 5.5%. Among the three triage strategies, AHPV genotyping with reflex LBC triage had the highest AUC value, which highlights the importance of HPV16/18 genotyping. Previous studies reported that the sensitivities of AHPV genotyping with reflex LBC triage were 84.6% (Wang et al., 2019) and 86.6% (Iftner et al., 2015) in the HPV-positive population. However, in this study, the sensitivity of AHPV genotyping with reflex LBC triage was relatively low (73.4%). First, AHPV-GT detects HPV16 and a subset of HPV18 and HPV45 cases. A summarized global meta-analysis indicated that HPV16 was the most frequently detected type; HPV18 ranked second place in CIN3 and invasive cervical cancer (ICC); HPV45 was more common than other non-HPV16/18 types in ICC (Guan et al., 2012). However, in China, HPV31/33/52/58 has a higher risk of HSIL+ than HPV18/45 in HPV-positive and cytology-negative women (Zhang et al., 2020a). Women with non-HPV16 18/45-positive and cytology-negative are followed up, which may be the reason for the low sensitivity of AHPV genotyping with reflex LBC triage. In addition, only 53.8% (458/851) of the AHPV-positive and LBC-negative women who needed to be referred for colposcopy actually had colposcopy. The low performance of colposcopy referral may also reduce the sensitivity of AHPV genotyping with reflex LBC triage. To summarize, AHPV genotyping with reflex LBC triage may be a feasible triage strategy for AHPV-based screening. However, in China, HPV extended genotyping is worth further study.

Excessive colposcopy referral not only wastes medical resources but also brings unnecessary mental burden to women. A good screening method should balance lesion detection and colposcopy referral. The number of colposcopes to be referred for each case of HSIL+ can be used as an indicator to measure the screening efficiency. In this study, the screening efficiency of AHPV (7.4, 749/101) is nearly equivalent to LBC (7.0, 519/74). The screening efficiency of AHPV genotyping with reflex LBC triage and AHPV-LBC triage was higher than that of the AHPV test alone. However, the detection rate of HSIL+ is also lower than that of AHPV primary screening. The requirements for medical resources and organization of each triage strategy are higher than those of AHPV primary screening. Therefore, AHPV screening has achieved a good balance in the detection of lesions and colposcopy referral, which is suitable for cervical cancer screening in middle-income areas.

This study has some limitations that need to be taken into consideration. First, 37% of women who needed to refer for colposcopy were not recalled. In future research, we should find ways to improve the colposcopy referral compliance of HPV-positive but cytology-negative women. Second, longitudinal follow-up should be carried out in women with both negative LBC and AHPV results. Finally, the performance of primary HPV screening with different triage strategies differed among age groups (Bao et al., 2022). Evaluation of the age-specific effectiveness of primary AHPV screening and possible triage strategies is warranted.

In conclusion, the HPV E6/E7 mRNA assay was found to be more sensitive than cytology and to have good specificity. This study suggests that primary screening with HPV E6/E7 mRNA assay is a candidate protocol suitable for cervical cancer screening in China. AHPV genotyping with reflex LBC triage may be a feasible triage strategy. Further longitudinal studies and extending genotyping studies are warranted for triage strategies in primary HPV screening.

## Data availability statement

The original contributions presented in the study are included in the article/supplementary material. Further inquiries can be directed to the corresponding authors.

## Ethics statement

This study was reviewed and approved by the Ethics Committee of Liaoning Cancer Hospital and Institute. The patients/participants provided their written informed consent to participate in this study. Written informed consent was obtained from the individual(s) for the publication of any potentially identifiable images or data included in this article.

## Author contributions

All the authors contributed to the conception and design of the study. JZ, DY, and XC organized the database. GL performed the statistical analysis. JZ wrote the first draft of the manuscript. JZ, DY, XC, GL, ZC, and CW contributed to the acquisition and analysis of data. All authors contributed to manuscript revision and read and approved the submitted version.

## Funding

This study was supported by the National Key Research and Development Program (2016YFC1303001).

## Acknowledgments

We would like to express our gratitude to the staff of the Department of Cancer Prevention and Treatment at Liaoning Cancer Hospital and Institute and community workers in Liaoning Province and Qingdao City.

## Conflict of interest

The authors declare that the research was conducted in the absence of any commercial or financial relationships that could be construed as a potential conflict of interest.

## Publisher’s note

All claims expressed in this article are solely those of the authors and do not necessarily represent those of their affiliated organizations, or those of the publisher, the editors and the reviewers. Any product that may be evaluated in this article, or claim that may be made by its manufacturer, is not guaranteed or endorsed by the publisher.
